# The lncRNA BDNF-AS/WDR5/FBXW7 axis mediates ferroptosis in gastric cancer peritoneal metastasis by regulating VDAC3 ubiquitination

**DOI:** 10.7150/ijbs.69454

**Published:** 2022-01-24

**Authors:** Guoquan Huang, Zhenxian Xiang, Haitao Wu, Qiuming He, Rongzhang Dou, Zaihuan Lin, Chaogang Yang, Sihao Huang, Jialin Song, Ziyang Di, Shuyi Wang, Bin Xiong

**Affiliations:** 1 Department of Gastrointestinal Surgery & Department of Gastric and Colorectal Surgical Oncology, Zhongnan Hospital of Wuhan University, Wuhan, China.; 2 Department of Gastrointestinal Surgery, Central Hospital of Enshi Tujia and Miao Autonomous Prefecture, Enshi, China.; 3 Hubei Cancer Clinical Study Center, Wuhan, China.

**Keywords:** BDNF-AS, FBXW7, VDAC3, ferroptosis, gastric cancer, peritoneal metastasis.

## Abstract

Ferroptosis is a novel form of cell death that is closely associated with the formation of many tumors. Our study focused on the mechanism by which long noncoding RNAs (lncRNAs) regulate ferroptosis in gastric cancer (GC) peritoneal metastasis (PM). We utilized lncRNA sequencing and protein profiling analysis to identify ferroptosis-associated lncRNAs and proteins. qRT-PCR was used to analyze the expression of BDNF-AS and FBXW7 in GC tissues and adjacent normal tissues. Chromatin isolation by RNA purification (ChIRP), RNA immunoprecipitation (RIP), chromatin immunoprecipitation (ChIP), and coimmunoprecipitation (co-IP) assays were performed to investigate the interaction between BDNF-AS and its downstream targets. Finally, the function of BDNF-AS was validated *in vivo* . We demonstrated that BDNF-AS was highly expressed in GC and PM tissues. High BDNF-AS expression was positively related to GC progression and poor prognosis. Functionally, BDNF-AS overexpression protected GC cells from ferroptosis and promoted the progression of GC and PM. Mechanistically, BDNF-AS could regulate FBXW7 expression by recruiting WDR5, thus affecting FBXW7 transcription, and FBXW7 regulated the protein expression of VDAC3 through ubiquitination. Conclusively, our research demonstrated that the BDNF-AS/WDR5/FBXW7 axis regulates ferroptosis in GC by affecting VDAC3 ubiquitination. BDNF-AS might be a biomarker for the evaluation of GC prognosis and the treatment of GC.

## Introduction

Gastric cancer (GC) is highly prevalent worldwide. Although some progress has been made in GC diagnosis and treatment in recent years, the clinical outcome of GC patients with liver, peritoneal or distant lymph node metastasis is very poor. Most patients survive for less than one year^1^-^3^. GC peritoneal metastasis (PM) accounts for 53% to 66% of all cases of distant metastasis^3^, ^4^. Some studies have indicated that the mechanism underlying the PM of GC might be associated with epigenetic changes, inactivation of tumor suppressor genes, expression of oncogenes, and failure of programmed cell death processes^5^, ^6^. However, the current understanding of the pathogenesis of PM is insufficient, and there is still a lack of effective means for diagnosing and treating PM.

Ferroptosis is a novel form of cell death that is different from cell apoptosis, necrosis, and autophagy. Ferroptosis mainly involves lethal reactive oxygen species (ROS) production and abnormal Fe^2+^ accumulation. Free Fe^2+^ is highly oxidized, and it easily undergoes a Fenton reaction with H_2_O_2_ to produce hydroxyl radicals that can cause oxidative damage to DNA, proteins and membrane lipids, promote the occurrence of lipid peroxidation, damage the cell membrane and lead to cell death^7^. The morphological features of ferroptosis include endoplasmic reticulum shrinkage, increased endoplasmic reticulum density, mitochondrial swelling, etc. 8. Zhang et al. demonstrated that miR-522 secreted by cancer-associated fibroblasts (CAFs) suppressed ferroptosis and promoted the progression of GC through the development of chemoresistance^9^. Based on the physiological characteristics of ferroptosis, it can be regulated by many molecular mechanisms and processes, such as epigenetic regulation, transcription or posttranscriptional regulation, and mitochondrial disorders^8^, ^10^. Among the many regulatory mechanisms, epigenetic modifications, including DNA methylation, histone modification, and long noncoding RNAs (lncRNAs), play vital roles in the process of ferroptosis^11^, ^12^. During tumorigenesis and metastasis, tumor suppressor genes are frequently affected by the local hypermethylation of CpG islands in their gene promoter regions that downregulate their expression levels, leading to tumor formation^13^. In mammals, approximately 70% of promoters include short CpG-dense sequences, and CpG-rich promoters are usually unmethylated and transcriptionally active; these promoters are dynamically regulated by methyltransferase and dioxygenase^14^. However, studies about how hypermethylation of the promoter regions of tumor suppressor genes leads to gene silencing and affects ferroptosis in gastric cancer metastasis are limited.

LncRNAs, crucial participants in epigenetics, generally lack protein-coding abilities and are often longer than 200 nt^15^. Emerging evidence has shown that lncRNAs can bind to DNAs, RNAs, and proteins to regulate gene expression in multiple manners, including at the epigenetic, transcriptional, and posttranscriptional levels^16^. Our previous study demonstrated that the expression level of lncRNA BDNF-AS was significantly increased in peritoneal cancer tissues compared with primary lesion tissues based on comprehensive analysis of a PM lncRNA microarray^17^. Recently, Lin et al. demonstrated that BDNF-AS could induce endocrine resistance and malignant progression through the RNH1/TRIM21/mTOR cascade in breast cancer^18^. However, the biological function of BDNF-AS in GC and GC PM has not been confirmed. The mechanism by which BDNF-AS promotes PM requires further study. Moreover, high-throughput protein analysis identified ferroptosis-related proteins named voltage-dependent anion channels 2 and 3 (VDAC2/VDAC3) in three pairs of GC and PM tissues. Further research found that VDAC3 was obviously overexpressed in PM tissues. Some studies have shown that VDAC3 can regulate the movement of molecules, ions and metabolites into and out of mitochondria 19. Furthermore, VDAC3 expression is upregulated in human malignant tumors, such as melanoma and thyroid tumors; VDAC3 is the binding site of the anticancer drug erastin, and overexpression of VDAC3 can increase sensitivity to erastin 8, 20-22. In melanoma, Yang et al. suggested that Nedd4 could ubiquitylate VDAC2/VDAC3 to suppress erastin-induced ferroptosis^8^. VDAC2 and VDAC3 are regulated by ubiquitination-mediated degradation. Our studies revealed that the VDAC3 protein is a substrate of FBXW7, which serves as a tumor suppressor in GC^23^, ^24^. In addition, FBXW7 can regulate apoptosis and ferroptosis in pancreatic cancer cells 25. However, the mechanisms by which FBXW7 regulates VDAC3 ubiquitination in GC remain unclear. Lin et al. confirmed that BDNF-AS could regulate the progression of breast cancer by regulating gene ubiquitination^18^, but no studies have been conducted on whether BDNF-AS participates in regulating ubiquitination in the progression of GC PM.

Considering that epigenetics, ubiquitination, and ferroptosis play vital roles in the PM of GC, we speculated that BDNF-AS plays a crucial role in the process of ferroptosis in advanced GC by regulating VDAC3 ubiquitination. In the present study, our results indicated that ferroptosis was significantly associated with the PM of GC. The expression level of BDNF-AS was significantly related to the poor prognosis of GC patients. Both *in vivo* and *in vitro* studies showed that GC cell ferroptosis decreased when BDNF-AS was overexpressed, which was conducive to the invasion and metastasis of GC. Further mechanistic research showed that the ubiquitination of VDAC3 was regulated through the BDNF-AS/WDR5/FBXW7 axis. Taken together, our study provides new insight into the role of ferroptosis in advanced GC, and these findings might be beneficial to scientific research and physicians.

## Methods and Materials

### Patients, tissues and ethics statement

We obtained 66 paired samples of pathologically confirmed GC tissues and adjacent normal tissues from patients who had not undergone treatment; the samples were obtained from the specimen bank of Cancer Institute of Zhongnan Hospital of Wuhan University from 2014 to 2016. All the samples were collected after informed consent was obtained from the patients and with the approval of the ethics committee of Zhongnan Hospital of Wuhan University (Wuhan, China; Ethical Approval Number: 2019079). In this study, five-year overall survival (OS) was defined as the proportion of patients who survived for five years after surgery and related treatments. Five-year progression-free survival (PFS) was defined the proportion of patients who survived for five years without disease progression after surgery and related treatments. PM in this study included peritoneal metastatic lesions found during surgery and postoperative follow-up.

### Cell line culture and reagents

Five GC cell lines (AGS, HGC-27, BGC-823, MGC-803 and MKN-45) and one human stomach epithelial cell line (GES-1) were obtained from the Cell Bank of Wuhan University. The cell lines were incubated in an incubator with 5% CO_2_ at 37 °C and were cultured in Dulbecco's modified Eagle medium (DMEM) (Gibco, USA) supplemented with 10% fetal bovine serum (FBS) and 2 mmol/L glutamine.

### Lentivirus, plasmids, and siRNA reagents and transfection experiments

The overexpression plasmid (GV367) carrying BDNF-AS (PO-BDNF-AS) and green fluorescent protein (GFP) was constructed by GenePharma Biotechnology Co. Ltd. (Shanghai, China); we packaged the lentivirus using the GM easy TM Lentiviral Packaging kit (Shanghai, China) and named the particles Lv-Oe-BDNF-AS and Lv-OeNC-BDNF-AS. Then, the BDNF-AS interference lentivirus (Lv-SH-BDNF-AS and Lv-SHNC-BDNF-AS), FBXW7 overexpression lentivirus (Lv-Oe-FBXW7 and Lv-OeNC-FBXW7), FBXW7 interference plasmid (SH-FBXW7 and SHNC-FBXW7) and WDR5 overexpression plasmid (PO-WDR5-1/2 and PONC-WDR5) were constructed by and purchased from GenePharma Biotechnology Co. Ltd. siRNA-WDR5-1/2 was constructed and synthesized by Jtsbio Co. Ltd. (Wuhan, China) and then transfected into MKN-45 cells, while PO-WDR5 was transfected into HGC-27 cells. Lipofectamine 2000 (Invitrogen, USA) was used in all transfection assays. Total RNA or protein was isolated at 24 or 48 h after transfection. Cell lines with stable overexpression or knockdown were established according to the Stable Cell Line Construction Manual, and stably transfected cell lines were obtained for subsequent experiments by further screening with puromycin (Sigma-Aldrich, USA) (the sequences of the relevant genes are listed in Supplementary table 1).

### Colony formation and wound healing assays

To investigate the proliferation ability of GC cells after BDNF-AS overexpression or knockdown, colony formation experiments were conducted. Cells were seeded in six-well plates at a density of 1,000 cells per well. Then, the cells were cultured in an incubator at 37 °C for two weeks and fixed with 4% paraformaldehyde. Finally, the cells were stained with 0.5% crystal violet, and corresponding images were captured using a microscope (Olympus-IX73, Japan).

Wound healing assays were performed to assess the migration ability of GC cells after the treatments. Briefly, cells were seeded in six-well plates, and when the cell density reached 90%, three wounds were created in the cell monolayer. Then, the cells were washed and incubated in an incubator at 37 °C, and images of the same area of the wounds were captured every 24 hours. Finally, the wound area was analyzed by ImageJ (NIH, USA).

### Transwell migration and invasion assays

The migration ability of GC cells after BDNF-AS overexpression or knockdown was evaluated using 24-well Transwell plates (8-μm pore size; Corning, USA) that were not coated with Matrigel (Falcon; BD Biosciences, USA). The invasion assay was performed utilizing 24-well Transwell plates (8-μm pore size; Corning, USA) that were precoated with Matrigel. In brief, 10^5^ cells were added to the upper chamber after resuspension in 0.5 ml serum-free DMEM, and 0.75 ml DMEM supplemented with 10% FBS was added to the lower chamber. After incubation for 48 h at 37 °C, we removed the remaining cells from the upper chamber, and the cells on the lower surface of the membrane were fixed with 4% paraformaldehyde and stained with crystal violet (0.5%). Finally, the cells were photographed and counted in four or five selected fields of view.

### Iron assay and transmission electron microscopy

Ferrous iron (Fe^2+^) and total iron levels were measured after BDNF-AS overexpression or knockdown using an iron assay kit (MAK025, Sigma USA). Cells were seeded in six-well plates (2×10^6^ cells per well) and treated with erastin, RSL3 and DMSO for 24 hours to establish a ferroptosis model. After collecting and washing the cells, the concentrations of Fe^2+^ and total iron were measured according to the protocol of the iron assay kit. Finally, the absorbance at 593 nm was measured using a multifunctional enzyme label analyzer (PE Enspire, USA). The remaining samples were utilized to observe the subcellular structure of GC cells after ferroptosis using transmission electron microscopy.

### Malondialdehyde (MDA) assay and glutathione (GSH) and glutathione disulfide (GSSG) assay

MDA, GSH and GSSG concentrations were measured using an MDA assay kit (A003-2, Nanjing China) and GSH assay kit (A061-2-1, Nanjing China), and the specific details of the procedure followed the manufacturer's protocol. Finally, the absorbance was measured using a multifunctional enzyme label analyzer (PE Enspire, USA).

### Lipid ROS assay

The lipid ROS levels of the treated cells was evaluated by using a lipid ROS kit (S0033S, Beyotime, Shanghai China) via flow cytometry (CytoFlex S, BECKMAN, USA). Lv-Oe-BDNF-AS-HGC-27 or Lv-SH-BDNF-AS-MKN-45 cells were seeded in six-well plates (2×10^6^ cells per well) and treated with erastin, RSL3 and DMSO for 24 hours. The ROS detection template was established, and the ROS levels were measured according to the manufacturer's protocol.

### Immunohistochemistry and reagents

GC specimens were fixed with 10% formaldehyde and then embedded in paraffin. After preparing 4-μm-thick continuous paraffin sections, immunohistochemistry was performed using anti-VDAC3 (1:200, Proteintech, USA), anti-WDR5 (1:200, Cell Signaling Technology, USA), and anti-FBXW7 (1:300, Proteintech, USA) antibodies. The results were obtained with an automatic digital slide scanning and analysis system (Aperio VERSA 8, Germany), and the immune response score (IRS) was calculated^26^.

### Subcellular fractionation and RNA fluorescence in situ hybridization (FISH) assays

GC cells (10^7^) were collected and washed twice with ice-cold phosphate-buffered saline (PBS). Nuclear and cytosolic fractionation assays were performed using a Nuclear and Cytoplasmic Extraction kit according to the manufacturer's protocol. Then, RNA was extracted from the nuclear fractions and cytoplasmic fractions by using the EASYspin Tissue and Cellular RNA Rapid Extraction kit (Aidlab, Beijing, China). Finally, qRT-PCR assays were performed to determine the relative expression of BDNF-AS in the nucleus and cytoplasm, and U6 and β-actin were used as the nuclear and cytoplasmic reference genes, respectively.

The FISH probe for BDNF-AS was purchased from GenePharma (Shanghai, China) (probe sequence: Supplementary table 1). According to the FISH kit (Servicebio, Wuhan), we first seeded MKN-45 cells in climbing slides and then fixed the cells with 4% paraformaldehyde. Next, the FISH assay was conducted. Finally, we analyzed the results and captured the images using a positive fluorescence microscope (Nikon, Japan).

### RNA extraction and quantitative real-time reverse transcription-polymerase chain reaction (qRT-PCR) assay

Total RNA was extracted from GC cell lines and tissues by using the EASYspin Tissue and Cellular RNA Rapid Extraction kit (Aidlab, Beijing, China) or TRIzol Reagent (Invitrogen, USA) according to the manufacturer's protocol. Then, after measuring the total RNA concentration of each sample with a Nanodrop 2000 ultramicroscopy spectrophotometer (Thermo Scientific, USA), 1 μg RNA was reverse-transcribed into cDNA following the instructions of the HiScript® Q RT SuperMix for qPCR (+gDNA wiper) kit (Vazyme, Nanjing, China). Next, qRT-PCR was performed on a Bio-Rad IQ5 Real-Time PCR instrument (Bio-Rad, USA) in a 20 µL reaction and utilizing SYBR-Green PCR Master Mix (Vazyme, Nanjing, China). The primers used in this experiment are shown in Table S1.

### Western blotting

Cells treated with the interference or overexpression constructs were washed with 1× PBS three times, and then, RIPA buffer supplemented with protease inhibitor (Thermo Scientific, USA) was added. The cell lysates were incubated in a 4 °C refrigerator for 30 minutes and ultrasonically lysed. Next, we quantified the protein level using a bicinchoninic acid (BCA) assay after denaturation of the proteins at 100 °C for 10 minutes. Total proteins were separated utilizing sodium dodecyl sulfate-polyacrylamide gels (SDS-PAGE) and then transferred to polyvinylidene fluoride (PVDF) membranes (Millipore, USA). After 2 hours of blocking with 5% skim milk, the primary antibody was added to the membranes and incubated at 4 °C overnight. Subsequently, horseradish peroxidase (HRP)-conjugated secondary antibodies were added and incubated for 1.5 h. Finally, the target proteins were detected with a Bio-Rad ChemiDoc XRS System. Band intensity was analyzed using Bio-Rad Image Lab software. The primary antibodies and dilution ratios used in this study were as follows: anti-FBXW7 (1:1000, Abcam, USA), anti-VDAC2/3 (1:1000, Proteintech, USA), anti-WDR5 (1:1000, Cell Signaling, USA), anti-GAPDH (1:5000, Santa Cruz, CA), anti-β-actin (1:1000, Proteintech, USA), anti-H3K27me2/3 (1:1000, ACTIVE MOTIF, USA), anti-H3K9me2/3 (1:1000, ACTIVE MOTIF, USA), anti-ubiquitin (rabbit polyclonal antibody; 1:1000, Proteintech, USA), anti-HA (1:1000, Proteintech, USA), anti-Flag (1:1000, Proteintech, USA), and anti-GFP (1:2000, Proteintech, USA). The concentration of the secondary antibodies was 1:5000.

### Bisulfite modification and quantitative methylation-specific PCR (Q-MSP)

The CpG island of the FBXW7 promoter region was predicted by MethPrimer2.0 (http://www.urogene.org/methprimer2/). Q-MSP was performed to detect the exact CpG island methylation rate of the FBXW7 promoter region using a Bio-Rad CFX96 instrument (USA)^27^. In this experiment, we divided the treated cells into five groups: GES-1, MKN-45 alone, PO-BDNF-AS-HGC-27, PO-BDNF-AS+PO-WDR5-HGC-27, and SH-BDNF-AS+si-WDR5-MKN-45. The primers were amplified by a fluorescence quantitative instrument, and the threshold cycle (Ct) value was obtained. The methylation rate was calculated according to the difference among the Ct values. Cmeth=100/[1+2^(cTCG-CTTG)] ×100% was used to calculate the methylation rate^28^. (FBXW7-promoter-Q-MSP-primers: Supplementary Table 1).

### Immunoprecipitation (IP) and ubiquitination assay

For IP, GC cells were cultured and treated with MG-132 (10 µM), HA-Ubi plasmid and other treatments related to the experiment. An IP assay was performed according to the protocol of the Co-IP kit (Abison Biotechnology Co. Ltd, China). The ubiquitination of VDAC3 was assessed by western blotting after obtaining the interacting protein. After incubation with the corresponding antibodies, band intensity was analyzed using Bio-Rad Image Lab software.

### Coimmunoprecipitation (co-IP) assay

Co-IP assays were carried out according to the protocol of the kit (Abison Biotechnology Co. Ltd, China). Western blotting was performed to detect and analyze the binding relationship between the FBXW7 and VDAC3 proteins.

### RNA-binding protein immunoprecipitation (RIP) assay

According to the protocol of the Magna RIP kit (Millipore, Billerica, MA, USA), cells were seeded in a plate (15 cm) and transfected with the relevant constructs to overexpress or knock down the target gene. The cells were harvested and lysed with RIP lysis buffer. The magnetic beads were washed with RIP wash buffer, and then, anti-WDR5 (5 µg, Cell Signaling, USA) and anti-IgG antibodies were added and incubated at room temperature for 30 minutes. Total RNA (10 μl, input control) was utilized as a control. Next, RIP was performed, and the samples were incubated in a refrigerator at 4 °C overnight. Subsequently, the incubated samples were washed 5 times with RIP wash buffer. Then, the RNA was extracted and purified by the TRIzol method, and the RNA concentration was measured by a Nanodrop 2000 ultramicroscopy spectrophotometer. Next, the RNA samples were reverse transcribed to cDNA, and expression levels were measured by qRT-PCR.

### Chromatin immunoprecipitation (ChIP) analysis

We cultured the cells and transfected them with BDNF-AS overexpression or knockdown constructs and designed PCR primers according to the binding sites of BDNF-AS and WDR5 or H3K27me3. According to the instructions of the Simple ChIP® Enzymatic Chromatin IP kit (Agarose Beads) (Cell Signaling, USA), cell culture cross-linking was performed, and the samples were prepared. Then, nuclear processing and chromatin splicing were performed. Next, the elution of chromatin from antibody/protein G agarose beads and reversal of the cross-linking were performed. Finally, the DNA was purified from the samples and subjected to qRT-PCR. The qRT-PCR products were identified via electrophoresis on a 2% agarose gel (ChIP primers: Supplementary Table 1).

### Chromatin isolation by RNA purification (ChIRP) assay

GC cells were cultured and collected into a 50-ml Falcon tube. Then, the ChIRP assay was conducted according to a reference book, the protocol of the ChIRP assay kit (Guangzhou, China) and the RiboTM ChIRP Probe instructions (Guangzhou, China). The ChIRP probe was designed by Ribo Biotech Ltd. The biotinylated BDNF-AS probe sequences are listed in Supplementary Table 1. The levels of the RNA and protein ChIRP products were detected by qRT-PCR and western blotting.

### Animal experiment

The animal experiments were approved by the Ethics Committee of Zhongnan Hospital of Wuhan University, and all the experiments were carried out in accordance with the “Wuhan University Laboratory Animal Care and Use Guidelines”. Four- to six-week-old nude mice (BALB/c) were purchased from Jiangsu Jicui Yaokang Biotechnology Co. Ltd. (Nanjing, China) and then randomly divided into 3 groups (n = 5 per group). PO-BDNF-AS-HGC-27 cells, PONC-BDNF-AS-HGC-27 cells, or PO-BDNF-AS+PO-FBXW7-HGC-27 cells (5×10^6^) were suspended in 100 μl DMEM and subcutaneously injected into the flanks of the mice in each group. After 10 days, we measured the tumor size every week using digital Vernier calipers and calculated the tumor volume based on the formula: volume = 1/2 × (width^2^×length). On the 30th day or when the tumor became larger than 1.5 cm in diameter, the mice were sacrificed. Subsequently, the subcutaneous graft tumors were removed for further experiments. For the intraabdominal tumor model, we randomly divided the mice into two groups (n = 6 per group). PO-BDNF-AS-HGC-27 cells or PONC-BDNF-AS-HGC-27 cells (8×10^6^) were suspended in 150 μL DMEM and intraperitoneally injected into the mice in each group (weight, approximately 18.0-19.0 g). Five weeks after injection, the mice were euthanized and necropsied to assess abdominal tumor burden and tumor location. Finally, we detected the mRNA and protein expression levels of relevant genes in the tumor tissues by RT-PCR and western blotting assays.

### Statistical analysis

SPSS (version 23.0, IBM SPSS, USA) and GraphPad Prism (version 8.0, GraphPad Software, USA) were used to analyze and visualize the results. To evaluate the relationship between BDNF-AS expression and FBXW7 expression, Pearson's correlation was performed. Then, we utilized the chi-square test to analyze the expression of BDNF-AS/FBXW7 and the clinicopathological features of the GC patients. Moreover, Kaplan-Meier curves and the log-rank test were used for survival analysis. Finally, the independent factors affecting the prognosis of GC patients were determined by using univariate and multivariate Cox proportional hazards regression models. All the cell experiments were performed at least three independent times. The results were considered statistically significant when the p value < 0.05.

## Results

### Identification and localization of BDNF-AS, which was highly expressed in GC and associated with poor prognosis of GC patients

**Figure 1:**
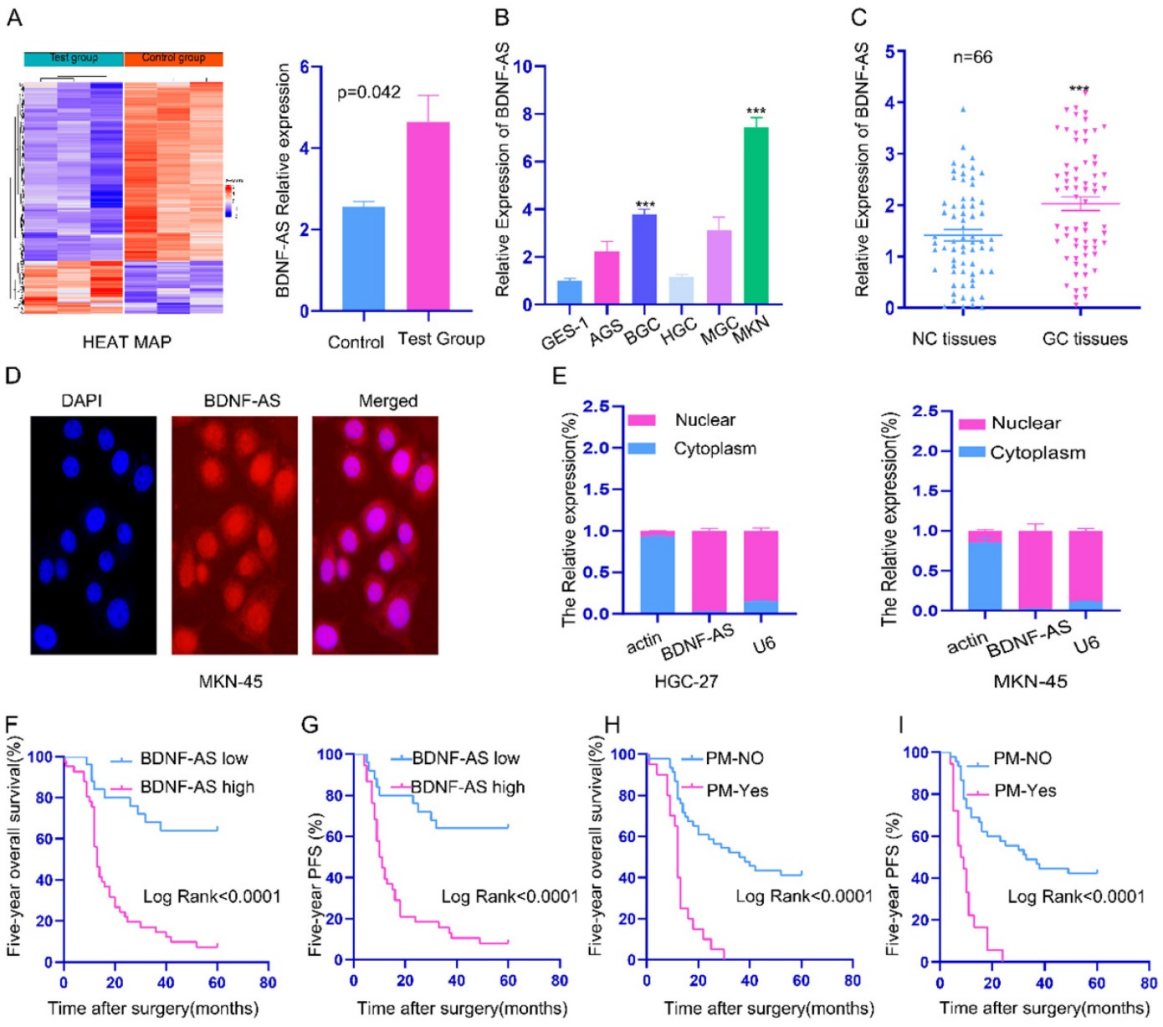
The expression, localization and clinicopathological significance of BDNF-AS in gastric cancer. *p < 0.05, **p < 0.01, ***p < 0.001, ****p < 0.0001. Data are shown as mean ± SEM (n = 3). (A): lncRNAs expression were performed in three pairs of GC and corresponding PM of GC tissues by microarray analysis and a heatmap of total lncRNA and a histogram of BDNF-AS was made according to the value of the samples and fluorescence intensity. **(B):** The relative expression of BDNF-AS in five GC cell lines and normal stomach epithelial cell line (GES-1) was detected by qRT-PCR assay. **(C):** The relative expression of BDNF-AS was detected by qRT-PCR assay in 66 pairs GC tissues and corresponding adjacent normal tissues.** (D):** The intracellular location of BDNF-AS was detected by RNA FISH assay (400ⅹ). **(E):** The cytoplasmic and nuclear distribution of BDNF-AS in HGC-27 and MKN-45 cell lines were identified using qRT-PCR assay. **(F-G):** The correlation with the expression level of BDNF-AS between five-year overall survival (F) and five-year progression-free survival (G) was analyzed using Kaplan-Meier survival analysis. **(H-I):** The correlation with peritoneal metastasis (PM) between five-year overall survival (H) and five-year progression-free survival (I) was analyzed using Kaplan-Meier survival analysis.

lncRNA microarray profiling showed that the expression of BDNF-AS in PM tissues was significantly higher than that in primary GC tissues (P=0.042) (Fig. 1A). Compared with that in the GES-1 cell line (a normal stomach mucosal cell line), the BDNF-AS mRNA expression level in 5 GC cell lines (AGS, HGC-27, BGC-823, MGC-803 and MKN-45) was higher (Fig. 1B). We further observed that the mRNA expression of BDNF-AS was significantly upregulated in GC tissues (p<0.01) (Fig. 1C). To determine the localization of BDNF-AS, FISH and subcellular fractionation assays were performed with the MKN-45 and HGC-27 cell lines, and we found that BDNF-AS was mainly localized in the nucleus (Fig. 1D, 1E).

Next, we explored the correlation between BDNF-AS expression and the clinicopathological parameters of GC patients (Table 1). The results revealed that the high expression of BDNF-AS was significantly correlated with lymph node metastasis, distant metastasis, peritoneal metastasis (PM) and tumor-node-metastasis (TNM) stage (P <0.05). However, there was no significant association with sex, age, cancer grade, cancer size, T stage, CEA, chemotherapy or CA199 (p>0.05). Further prognostic survival analysis indicated that patients with high BDNF-AS expression had much shorter 5-year overall survival (OS) (Fig. 1F) and 5-year progression-free survival (PFS) times (Fig. 1G). Univariate and multivariate analyses showed that BDNF-AS expression was significantly associated with poor PFS (HR: 3.989, 95% CI: 1.629 - 9.767, p=0.002) and OS (HR: 4.036, 95% CI: 1.663 - 9.796, P=0.002) (Table 2). These data indicated that BDNF-AS was closely related to the progression, metastasis and prognosis of GC, BDNF-AS can be used as an independent indicator of prognosis.

### BDNF-AS was associated with ferroptosis in GC cells via VDAC3

**Figure 2:**
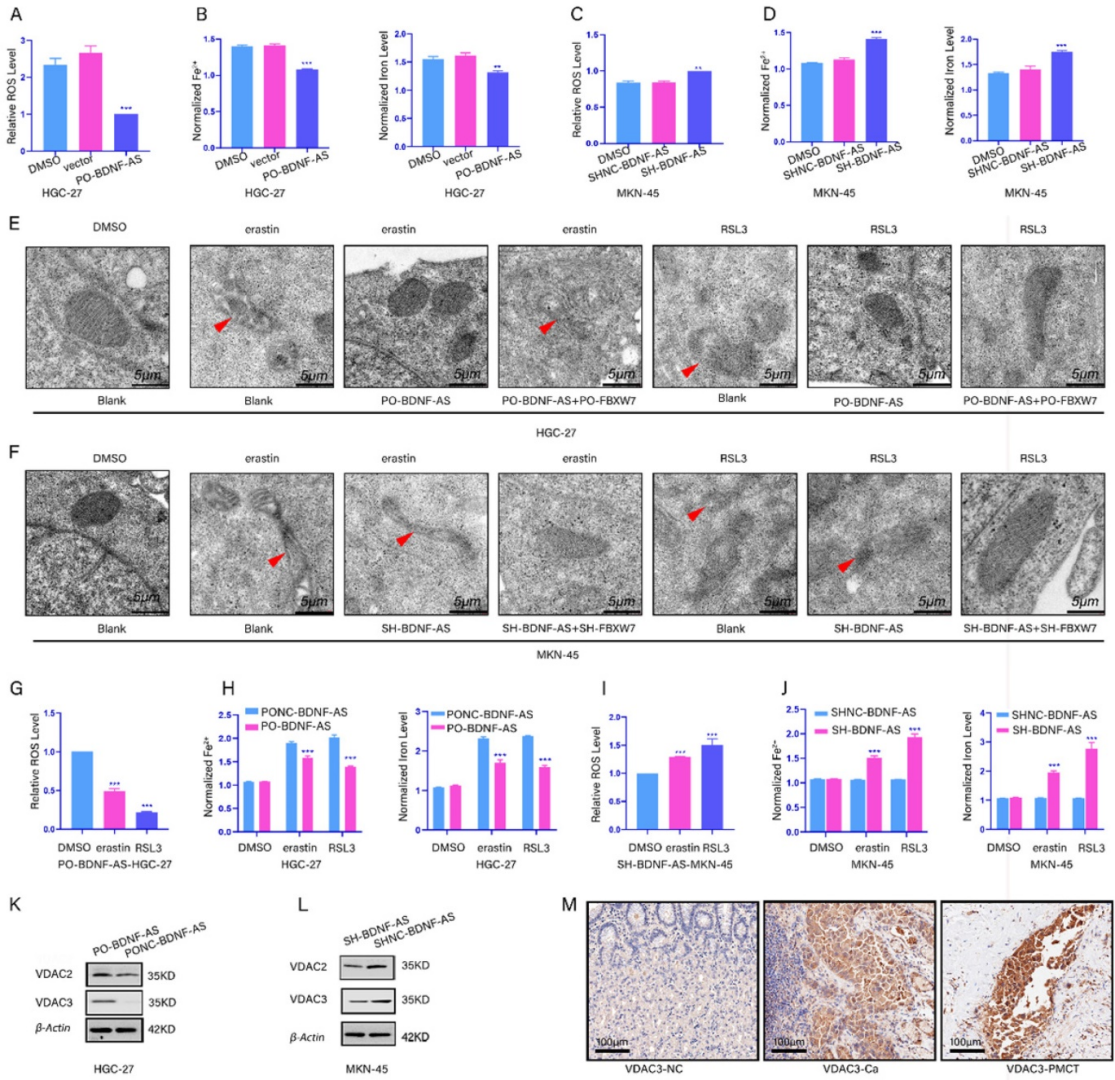
BDNF-AS was associated with ferroptosis in gastric cancer cells. *p < 0.05, **p < 0.01, ***p < 0.001, ****p < 0.0001. Data are shown as mean ± SEM (n = 3). (A): The level of ROS after overexpression with BDNF-AS was detected by flow cytometry in HGC-27, DMSO served as a control.** (B):** The levels of Fe^2+^ and Iron after overexpression with BDNF-AS were detected by Iron assay kit in HGC-27 cells, DMSO served as a control. **(C):** The level of ROS after knockdown with BDNF-AS was detected by flow cytometry in MKN-45, DMSO served as a control. **(D):** The levels of Fe^2+^ and Iron after knockdown with BDNF-AS were detected by Iron assay kit in MKN-45, DMSO served as a control. **(E):** Transmission electron microscopy of HGC-27 cells treated with DMSO (24hrs), erastin (5 μM, 24hrs), RSL3 (1 μg/ml, 24hrs), PO-BDNF-AS+erastin (5 μM, 24hrs), PO-BDNF-AS+RSL3 (1 μg/ml, 24hrs). Single red arrowhead: shrunken mitochondria and mitochondrial cristae disappeared. **(F):** Transmission electron microscopy of MKN-45 cells treated with DMSO (24hrs), erastin (5 μM, 24hrs), RSL3 (1 μg/ml, 24hrs), SH-BDNF-AS+erastin (5 μM, 24hrs), SH-BDNF-AS+RSL3 (1 μg/ml, 24hrs). Single red arrowhead: shrunken mitochondria and mitochondrial cristae disappeared.** (G):** The level of ROS after overexpression with BDNF-AS and treatment with DMSO, erastin (5 μM) and RSL3 (1 μg/ml) for 24h was detected by flow cytometry in HGC-27 cells. **(H):** The levels of Fe^2+^ and Iron after overexpression with BDNF-AS and treatment with DMSO, erastin (5 μM) and RSL3(1 μg/ml) for 24h were detected by Iron assay kit in HGC-27 cells.** (I):** The level of ROS after knockdown with BDNF-AS and treatment with DMSO, erastin (5 μM) and RSL3 (1 μg/ml) for 24h were detected by flow cytometry in MKN-45 cells.** (J):** The levels of Fe^2+^ and Iron after interference with BDNF-AS and treatment with DMSO, erastin (5 μM) and RSL3 (1 μg/ml) for 24h were detected by Iron assay kit in MKN-45 cells. **(K-L):** The variations of VDAC2/VDAC3 proteins expression level of overexpression (K) or knockdown (L) BDNF-AS were determined by western blotting assay in HGC-27 and MKN-45 cells.** (M):** The representative IHC staining images for VDAC3 in the para-cancerous normal tissues, GC tissues and PM of GC tissues.

To reveal the relationship between BDNF-AS and ferroptosis, we constructed with stable BDNF-AS overexpression or knockdown cell lines via lentiviral transduction. Next, qRT-PCR was performed to measure the overexpression or knockdown efficiency (Supplementary figure-1A, 1B). Then, we measured the changes in the concentrations of ROS, Fe^2+^, and total iron. We found that the concentrations of ROS, Fe^2+^ and total iron were decreased after the overexpression of BDNF-AS; however, after knocking down BDNF-AS expression, the concentrations of ROS, Fe^2+^ and total iron were increased (Fig 2A-2D). Then, we established a ferroptosis model using erastin and RSL3. The characteristics of ferroptosis in HGC-27 or MKN-45 cells were observed by transmission electron microscopy. The results showed that compared with DMSO-treated cells, cells treated with erastin and RSL3 exhibited unique morphological characteristics associated with ferroptosis, and these results are consistent with the existing literature^22^. We also found that ferroptosis was decreased after BDNF-AS overexpression and increased after BDNF-AS knockdown when erastin and RSL3 were incubated with BDNF-AS-overexpressing or BDNF-AS knockdown cells for 24 hours (Fig. 2E, 2F). The results of ROS, iron, MDA, GSH, and GSSG assays indicated that BDNF-AS overexpression could reduce the concentrations of ROS (Fig. 2G), iron and Fe^2+^ (Fig. 2H), MDA (Supplementary figure-1C), and GSSG (Supplementary figure-1D) and increase the concentration of GSH (Supplementary figure-1E). BDNF-AS knockdown promoted the ferroptosis of MKN-45 cells, which was mainly manifested in the following aspects: the concentrations of ROS (Fig. 2I), iron and Fe^2+^ (Fig. 2J), MDA (Supplementary figure-1F), and GSSG (Supplementary figure-1G) were increased, and the expression of GSH was decreased (Supplementary figure-1H). Interestingly, these results indicated that the change in ferroptosis marker levels induced by erastin was more significant than that induced by RSL3. Thus, we explored the mechanism by which BDNF-AS functions in GC ferroptosis. Existing evidence has demonstrated that erastin can induce ferroptosis by binding to VDAC2/VDAC3^29^. Furthermore, our protein profile results of GC and PM tissues indicated that VDAC2/VDAC3 were abnormally expressed in GC and PM samples (Supplementary figure-1I). Then, we examined the changes in the VDAC2/VDAC3 protein levels after the overexpression or knockdown of BDNF-AS to identify the relationship between BDNF-AS expression and ferroptosis. The results suggested that compared with VDAC2, the VDAC3 protein levels were significantly increased or decreased when BDNF-AS was overexpressed or knocked down, respectively (Fig. 2K, 2L). Moreover, the expression of VDAC3 was measured by immunohistochemistry, which further supported these results (Fig. 2M). The above data indicated that BDNF-AS expression was correlated with ferroptosis in GC and that the associated mechanism might be closely related to the VDAC3 protein.

### FBXW7 could regulate VDAC3 protein expression through ubiquitination

**Figure 3:**
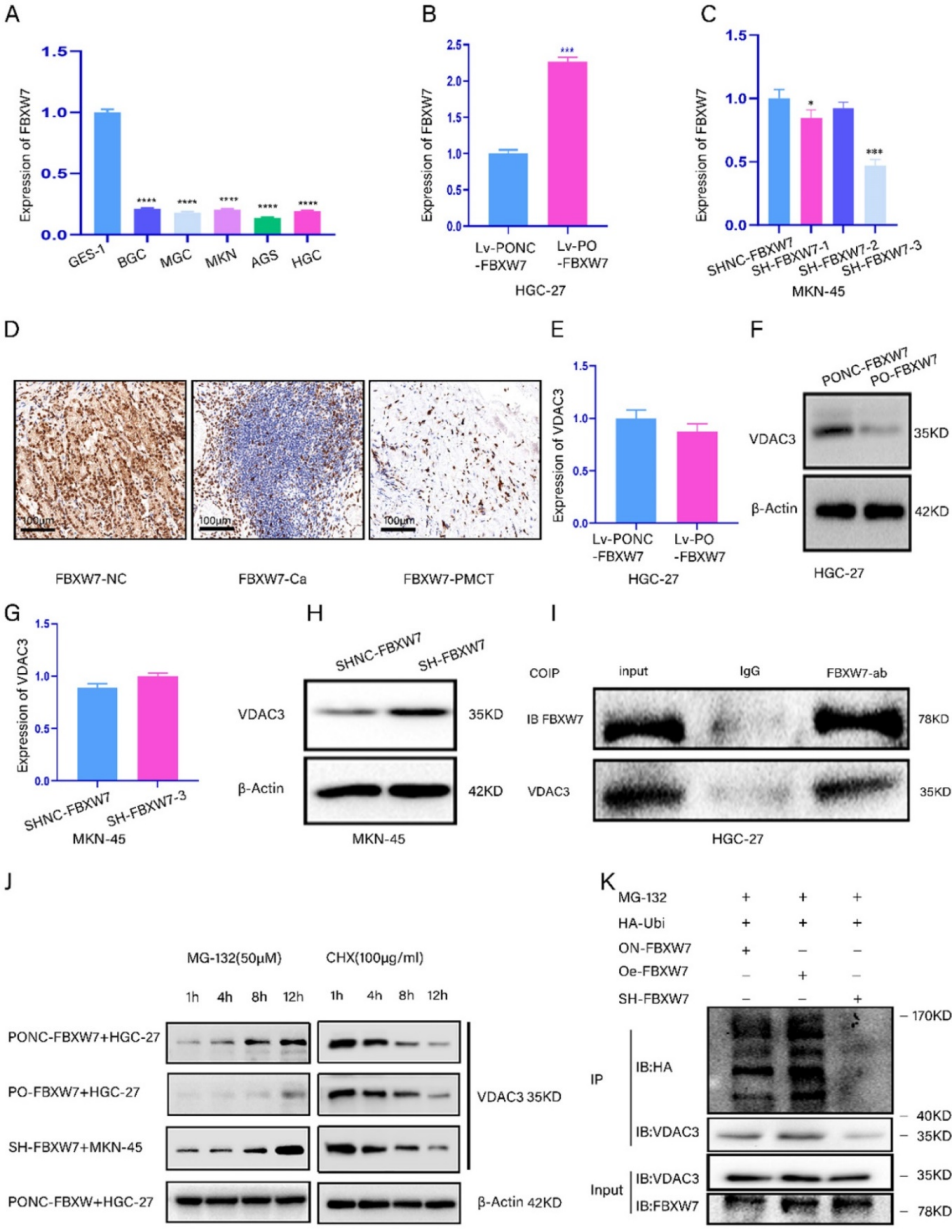
FBXW7 could regulate VDAC3 through ubiquitination. *p < 0.05, **p < 0.01, ***p < 0.001, ****p < 0.0001. Data are shown as mean ± SEM (n = 3). (A): The relative expression of FBXW7 in five GC cell lines and normal stomach epithelial cell line (GES-1) was detected by qRT-PCR assay. **(B):** The expression efficiency of lentivirus-mediated FBXW7 overexpression and negative control (NC) was detected in HGC-27 cell line using qRT-PCR assay. **(C):** The expression efficiency of plasmid-mediated FBXW7 knockdown containing SH-FBXW7-1, SH-FBXW7-2, SH-FBXW7-3 and negative control (NC) was detected in MKN-45 cell line using qRT-PCR assay. **(D):** The representative IHC staining images for FBXW7 in the para-cancerous normal tissues, GC tissues and GS PM tissues. **(E):** The mRNA expression level of VDAC3 after FBXW7 overexpression in HGC-27 cells was detected by qRT-PCR assay. **(F):** The protein expression level of VDAC3 after FBXW7 overexpression in HGC-27 cells was detected by western blotting assay. (G): The mRNA expression level of VDAC3 after FBXW7 knockdown in the MKN-45 cells was detected by qRT-PCR assay. (H): The protein expression level of VDAC3 after FBXW7 knockdown in the MKN-45 cells was detected by western blotting assay. **(I):** The interaction between FBXW7 and VDAC3 was identified in HGC-27 cells by coimmunoprecipitation(co-IP). SDS-PAGE separated the immunoprecipitates of input (20%) and VDAC3. western blotting was performed to confirm the interaction between FBXW7 and VDAC3. **(J):** Cell groups were treated with the proteasome inhibitor MG-132 (50μM) or the protein-synthesis inhibitor cycloheximide CHX (100μg/ml) at different time points (1h, 4h, 8h, 12h), and the change of VDAC3 protein at different time points was detected by western blotting assay. **(K):** HGC-27 or MKN-45 cells were treated with MG-132(50μM) and transfected with HA-ubi and simultaneously overexpression or knockdown FBXW7, FBXW7 vector served as a negative control. Cell lysates were immunoprecipitated with anti-VDAC3 antibody to identified ubiquitination of VDAC3 with anti-HA antibody using western blotting assay.

We aimed to demonstrate that FBXW7 regulated VDAC3 expression through ubiquitination. We detected the expression of FBXW7 in five GC cell lines and the GES-1 cell line (Fig. 3A). Then, the efficiency of FBXW7 overexpression and knockdown was confirmed by qRT-PCR (Fig. 3B, 3C). Furthermore, the results of the IHC assay showed that the expression level of FBXW7 was the lowest in peritoneal metastasis tissues (Fig. 3D). Moreover, we found that the mRNA level of VDAC3 was not significantly different, while the protein expression level of VDAC3 was significantly changed in HGC-27 or MKN-45 cells in which FBXW7 was overexpressed or knocked down (Fig. 3E-3H). Moreover, the co-IP results showed that FBXW7 interacted with VDAC3 (Fig. 3I). The above results indicated that FBXW7 might interact with VDAC3 at the posttranscriptional level. To further investigate whether VDAC3 was degraded by the ubiquitination pathway, we treated GC cells in which FBXW7 was overexpressed or knocked down with cycloheximide (CHX) (a protein synthesis inhibitor) and MG-132 (a specific proteasome inhibitor). The results indicated that MG-132 could abolish the downregulation of VDAC3 protein expression in FBXW7-overexpressing GC cells (Fig. 3J). These results indicated that the ubiquitin-proteasome pathway might be required for FBXW7-mediated degradation of the VDAC3 protein. To confirm that the level of VDAC3 ubiquitination was affected by FBXW7, we conducted an IP assay and ubiquitination experiment after treating GC cells with MG-132 (50 μM), HA-Ubi plasmid and FBXW7 overexpression or knockdown constructs. The western blotting results showed that the level of VDAC3 ubiquitination was increased after FBXW7 overexpression compared with the negative control. However, the level of VDAC3 ubiquitination was decreased after FBXW7 knockdown (Fig. 3K). In conclusion, we found that FBXW7 could ubiquitylate the VDAC3 protein.

### BDNF-AS downregulated the expression of FBXW7 and promoted the growth of GC cells by inhibiting FBXW7

**Figure 4:**
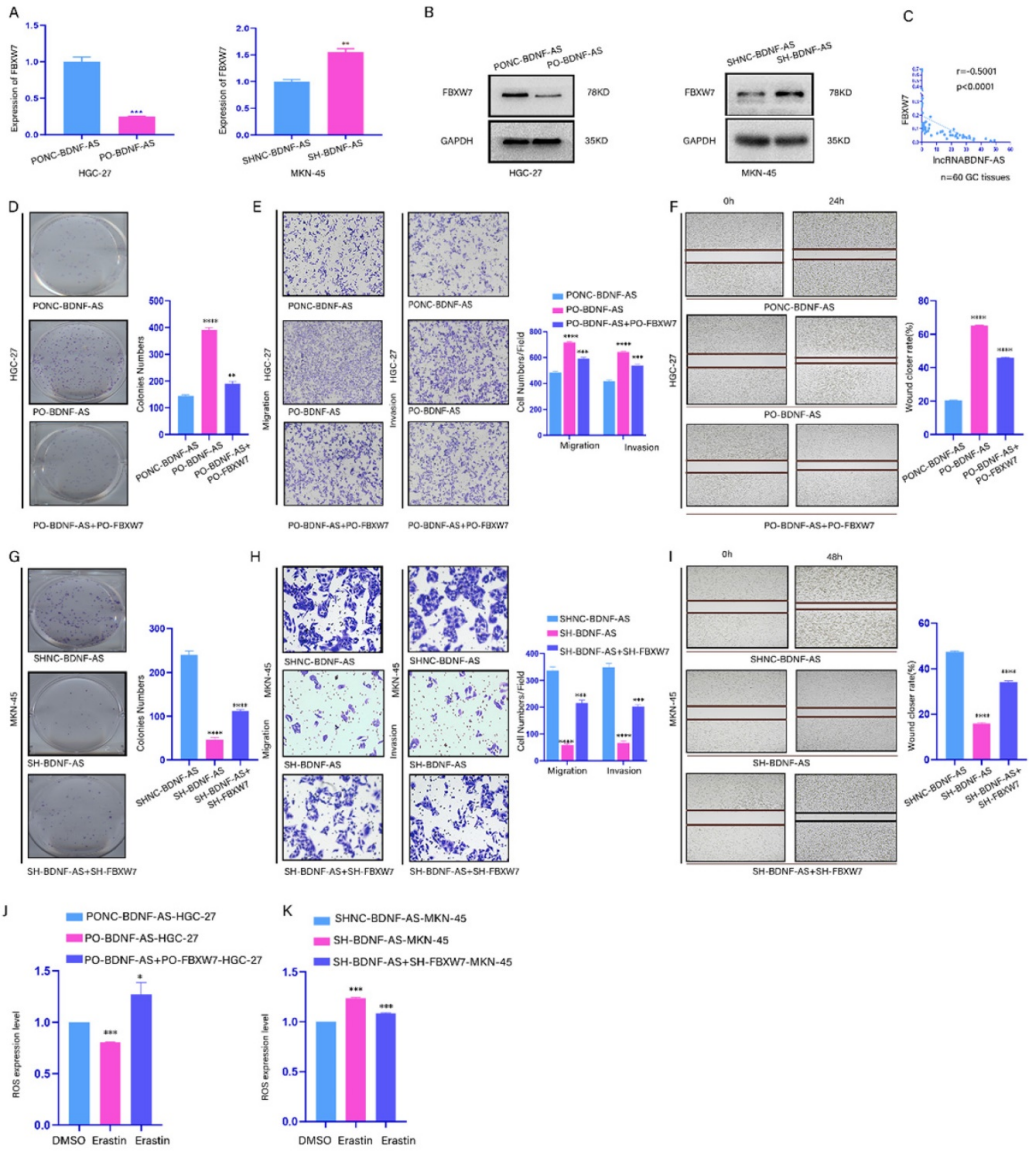
BDNF-AS downregulated the expression of FBXW7 and promoted the growth of GC cells by inhibiting FBXW7.Representative images of migratory or invaded cells (magnification, ×200) were shown. *p < 0.05, **p < 0.01, ***p < 0.001, ****p < 0.0001. Data were shown as mean ± SEM (n = 3). (A): The variation of FBXW7 mRNA expression level was detected by qRT-PCR assay after BDNF-AS overexpression or knockdown in HGC-27 and MKN-45 cells. **(B):** The variation of FBXW7 protein expression level was detected by western blotting after BDNF-AS overexpression or knockdown in HGC-27 and MKN-45 cells. **(C):** The mRNA expression levels of BDNF-AS and FBXW7 were obvious negative association in GC patients using pearson correlation analysis (Rs=-0.5001, p<0.0001). **(D-F):** Cell proliferation, invasion and migration ability of GC cells (HGC-27) negative control or treatment with BDNF-AS (PO-BDNF-AS or PO-BDNF-AS+PO-FBXW7) were detected by the colony formation (D), transwell (E) and wound healing assay (F) (magnification, 200×). **(G-I):** Cell proliferation, invasion and migration ability of GC cells (MKN-45) negative control or treatment with BDNF-AS (LV-SH-BDNF-AS or Lv-SH-BDNF-AS+SH-FBXW7) were detected by the colony formation (G), transwell (H) and wound healing assay (I) (magnification, 200×). **(J):** The level of ROS was detected by flow cytometry after BDNF-AS overexpression or combined overexpression of BDNF-AS and FBXW7 in erastin-treated (5 μM) GC cells (HGC-27) for 24 hours. DMSO served as a negative control.** (K):** The level of ROS was detected by flow cytometry after BDNF-AS knocked down or combined knockdown of BDNF-AS and FBXW7 in erastin-treated (5 μM) GC cells (MKN-45) for 24 hours. DMSO served as a negative control.

To explore whether BDNF-AS can regulate FBXW7 expression, the mRNA and protein expression levels of FBXW7 were assessed in GC cells in which BDNF-AS was overexpressed or knocked down (Fig. 4A, 4B). Then, the mRNA expression levels of FBXW7 and BDNF-AS were measured in 60 GC tissues by qRT-PCR (Fig. 4C). Our data indicated that the BDNF-AS levels and the FBXW7 levels were negatively correlated. Moreover, to further confirm this regulatory relationship, colony formation, Transwell and wound healing assays were conducted with Lv-Oe-BDNF-AS-HGC-27 or Lv-SH-BDNF-AS-MKN-45 cells, and the results showed that the proliferation, migration, invasion and metastasis of the cells were increased after the overexpression of BDNF-AS. In contrast, the opposite results were observed when BDNF-AS was knocked down. Then, we conducted rescue experiments in BDNF-AS overexpression or knockdown cells by overexpressing or knocking down FBXW7, and the results described above could be partially rescued by the overexpression or knockdown of FBXW7 (Fig. 4D-4I). Moreover, we assessed the changes in ROS production in BDNF-AS overexpression or BDNF-AS knockdown cells after FBXW7 overexpression or knockdown and treatment with erastin or DMSO. The results showed that the changes in ROS levels could be partly attenuated by the overexpression or knockdown of FBXW7 (Fig. 4J, 4K). In conclusion, our data suggested that BDNF-AS could downregulate the expression of FBXW7 in GC cells to exert its biological effects.

### BDNF-AS could regulate FBXW7 expression by recruiting WDR5

**Figure 5:**
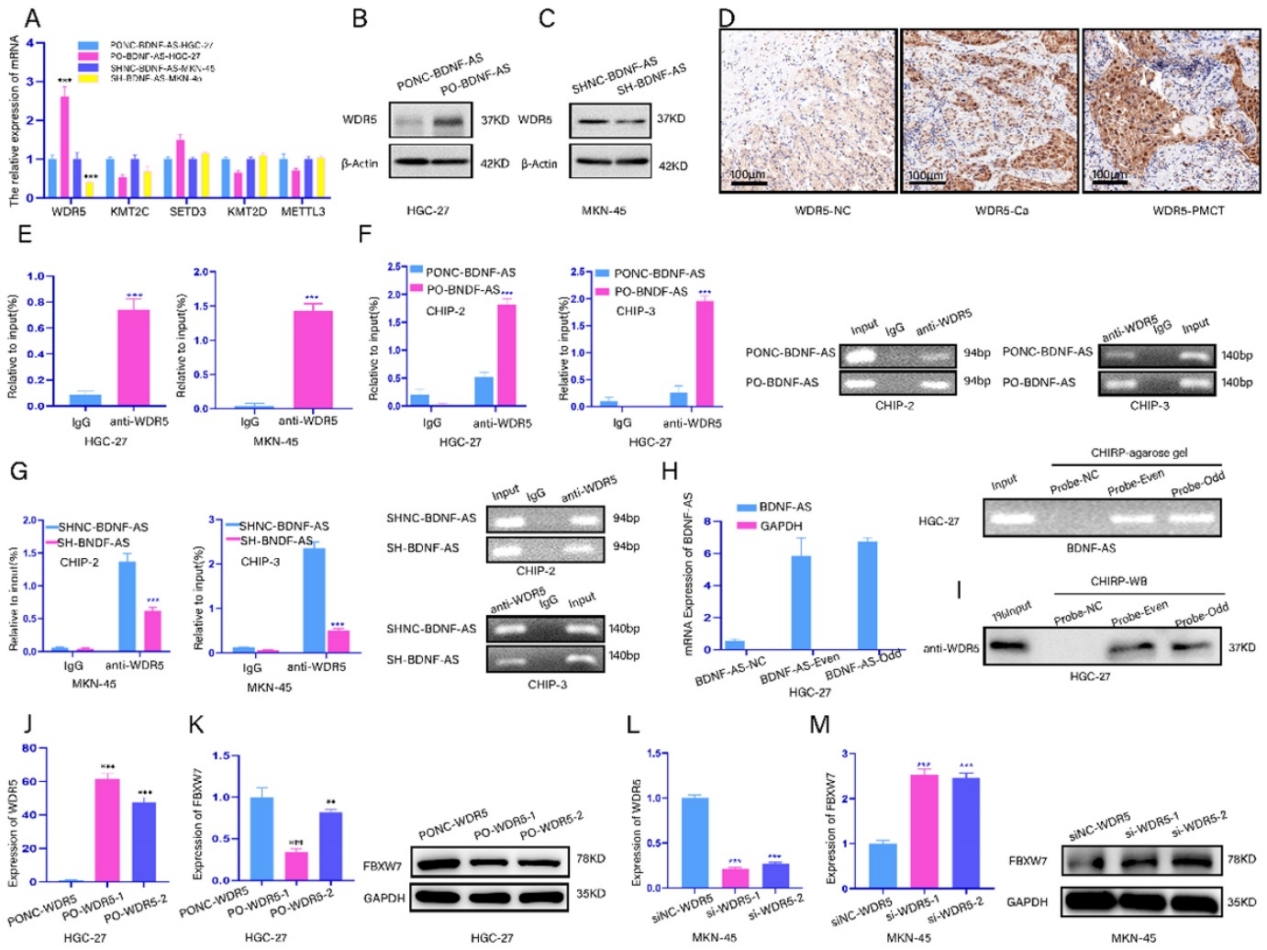
BDNF-AS could regulate FBXW7 expression by recruiting WDR5. Representative images of migratory or invaded cells were shown. *p < 0.05, **p < 0.01, ***p < 0.001, ****p < 0.0001. Data were shown as mean ± SEM (n = 3). (A): The mRNA expression changes of WDR5, KMT2C, SETD3, KMT2D and METTL3 genes were detected after BDNF-AS overexpression or interference in the HGC-27 or MKN-45 cell line by qRT-PCR assay. **(B-C):** The protein expression levels of WDR5 after BDNF-AS overexpression or knockdown was determined in HGC-27 or MKN-45 cells by western blotting assay. **(D):** The representative IHC staining images for WDR5 in the para-cancerous normal tissues, GC tissues and GS PM tissues. **(E):** RNA immunoprecipitation (RIP) assay confirmed the direct binding relationship between BDNF-AS and WDR5 in the HGC-27 and MKN-45 cells. RIP product RNAs were detected by qRT-PCR for BDNF-AS, the fold enrichment of BDNF-AS was relative to its corresponding IgG control. **(F-G):** ChIP assay analyzed the input (2%), IgG and WDR5 status in the HGC-27 or MKN-45 cells after BDNF-AS knockdown or overexpression. The primers in the ChIP experiment included three binding sites of BDNF-AS and WDR5 and named CHIP-1 (Supplementary Figure-1K,1L), CHIP-2 (F) and CHIP-3 (G). The CHIP products demonstrated the direct binding relationship between BDNF-AS and WDR5 by qRT-PCR assay. The products of qRT-PCR were determined by 2% agarose gel and the values were normalized to input (2%). **(H):** The enrichment of BDNF-AS in both even and odd probe groups targeting BDNF-AS relative to NC probe in HGC-27 cells, as detected by ChIRP assay. GAPDH served as a negative control. The products of qRT-PCR were determined by 2% agarose gel and the values were normalized to input (2%). **(I):** The enrichment of WDR5 protein in both even and odd probe groups targeting BDNF-AS relative to the NC probe in HGC-27 cells, as detected by ChIRP assay and western blotting. **(J):** The expression efficiency of WDR5 overexpression containing PO-WDR5-1, PO-WDR5-2, and negative control (NC) was detected in HGC-27 cell line using qRT-PCR assay. **(K):** The mRNA and protein expression levels of FBXW7 after WDR5 overexpression (PO-WDR5-1, PO-WDR5-2) were determined in HGC-27 cells by qRT-PCR and western blotting assay, WDR5 vector served as a negative control. **(L):** The expression efficiency of WDR5 knockdown containing si-WDR5-1, si-WDR5-2 and negative control (NC) was detected in MKN-45 cell line using qRT-PCR assay. **(M):** The mRNA and protein expression levels of FBXW7 after WDR5 knockdown (si-WDR5-1, si-WDR5-2) and negative control group were determined in MKN-45 cells by qRT-PCR and western blotting assay.

FBXW7 methylation and histone methylation are mainly related to WDR5, METTL3, SETD3, KMT2D and KMT2C^30^-^34^. Thus, we screened these five enzymes after the overexpression or knockdown of BDNF-AS by qRT-PCR. The results showed that the mRNA level of WDR5 was dramatically increased or decreased after BDNF-AS overexpression or knockdown, respectively. Additionally, when BDNF-AS was overexpressed or knocked down, the protein expression of WDR5 exhibited the same changes (Fig. 5A-C). Next, we validated the expression of WDR5 in five GC cell lines and GES-1 cell lines by qRT-PCR Supplementary figure 1J), and the protein expression of WDR5 in normal gastric tissues, GC tissues and GC PM tissues was measured by immunohistochemistry (Fig. 5D). We found that WDR5 was more highly expressed in GC cell lines, GC tissues and GC PM tissues than in normal controls.

Next, we investigated whether BDNF-AS could recruit WDR5. We found that BDNF-AS might bind to WDR5 according to RNA-Protein Interaction Prediction (RPISeq). Then, a RIP assay was performed to verify whether BDNF-AS bound to WDR5 in GC cell lines (HGC-27 or MKN-45). We observed more significant enrichment of WDR5 with the anti-WDR5 antibody than with IgG (Fig. 5E). ChIP assays were utilized to find direct evidence that BDNF-AS associates with WDR5, and the results showed that BDNF-AS could directly bind to WDR5 at chip-site 2 and chip-site 3 but not IgG, especially when BDNF-AS was overexpressed; however, BDNF-AS did not bind to WDR5 at chip-site 1. The products were identified by agarose gel electrophoresis (Fig. 5F, 5G) (Fig. chip-site 1 SUP-K; primer information: Supplementary Table 1. Furthermore, we identified the interaction between BDNF-AS and WDR5 through ChIRP. We divided BDNF-AS probes into even and odd groups to create two independent probe sets. Compared to the nontargeting control probe, both even and odd probe sets pulled down most of the BDNF-AS molecules from HGC-27 cells, as shown by the qRT-PCR results; the products were confirmed by agarose gel electrophoresis (Fig. 5H). In addition, we observed significant enrichment of WDR5 in the groups of even and odd probes targeting BDNF-AS relative to the normal control probe by detecting the protein products of ChIRP by western blotting (Fig. 5I). These data revealed that BDNF-AS could recruit and bind to the WDR5 protein.

Subsequent experiments aimed to explore whether WDR5 acts on FBXW7. The mRNA levels of WDR5 overexpression and knockdown were detected by qRT-PCR in GC cells (Fig. 5J, Fig 5L). Then, we found that the mRNA and protein levels of FBXW7 were downregulated when WDR5 expression was overexpressed (Fig. 5K). However, when WDR5 was knocked down, the mRNA and protein levels of FBXW7 exhibited the exact opposite trends (Fig. 5M). These results demonstrated that WDR5 could negatively regulate the expression of FBXW7. In conclusion, our data indicated that BDNF-AS could regulate FBXW7 expression by recruiting WDR5.

### The BDNF-AS/WDR5/FBXW7 axis could regulate the ferroptosis of GC by acting on the VDAC3 protein

**Figure 6:**
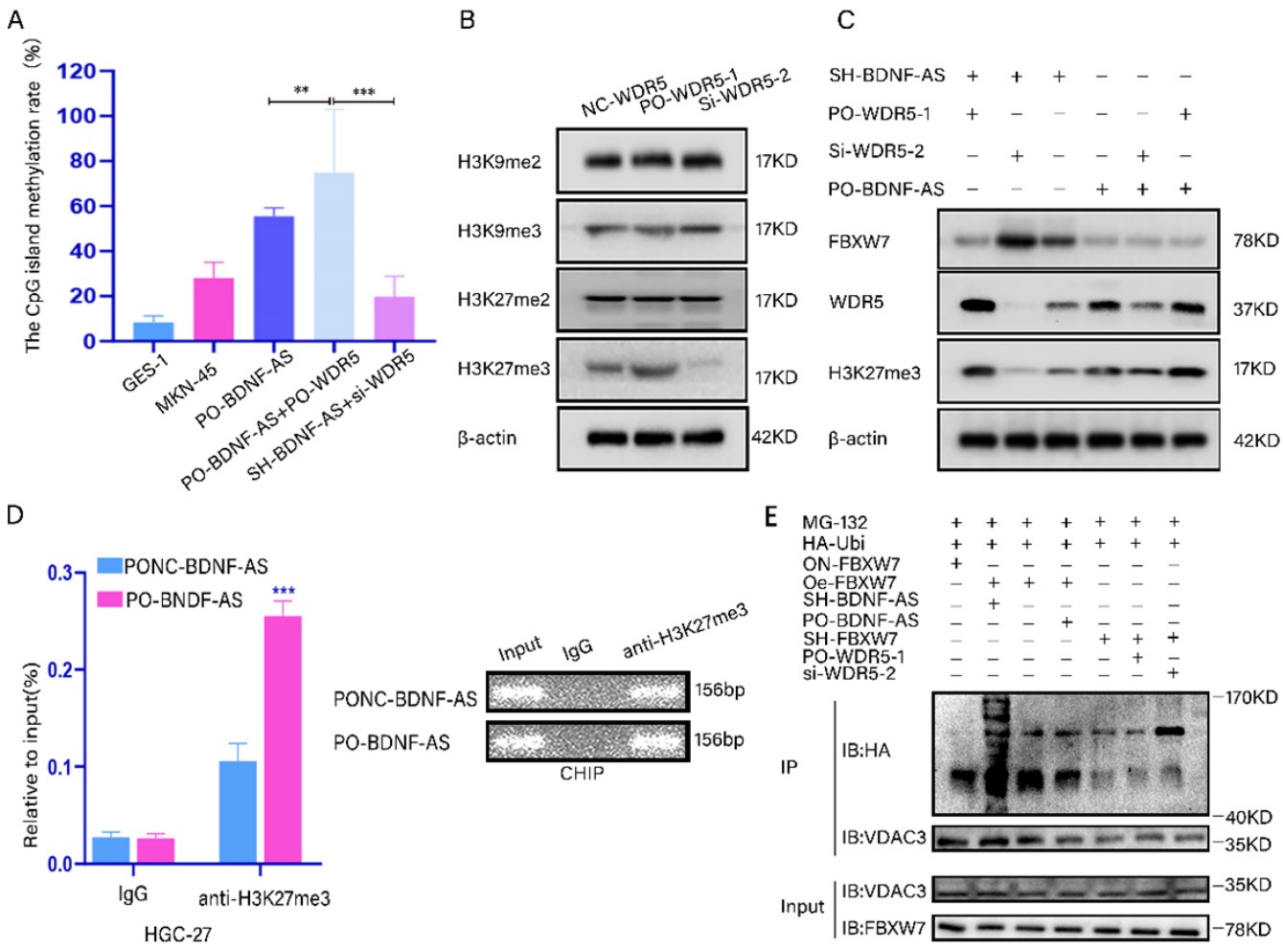
The BDNF-AS/WDR5/FBXW7 axis could regulate the ferroptosis of GC by acting on the VDAC3 proteins. *p < 0.05, **p < 0.01, ***p < 0.001, ****p < 0.0001. Data were shown as mean ± SEM (n = 3). (A): the CpG island methylation levels of FBXW7 promoter region in the treated GC cells (PO-BDNF-AS, PO-BDNF-AS+PO-WDR5, SH-BDNF-AS+si-WDR5) was determined by Q-MSP assay, GES-1 and MKN-45 cells served as negative control. **(B):** The protein expression levels of H3K9me2, H3K9me3, H3K27me2 and H3K27me3 were determined by western blotting in WDR5 overexpression and knockdown cells. **(C):** The protein expression level changes of FBXW7, WDR5 and H3K27me3 were detected by western blotting in the BDNF-AS and WDR5 overexpression or knockdown corresponding treated GC cells. **(D):** ChIP assay analyzed the input (2%), IgG and H3K27me3 status of candidate BDNF-AS target gene FBXW7 promoter region in the HGC-27 cells after BDNF-AS overexpression. The CHIP product demonstrated the enrichment level of H3K27me3 by qRT-PCR assay. The product of qRT-PCR was determined by 2% agarose gel and the values were normalized to input (2%). **(E):** HGC-27 or MKN-45 cells were treated with BDNF-AS, WDR5 and FBXW7 overexpression or knockdown accordingly, and simultaneously transfected HA-Ubi plasmid and treated with MG-132 (10 mM). Cell lysates were immunoprecipitated with anti-VDAC3 antibodies to identified polyubiquitination of VDAC3 with anti-HA antibodies using western blotting.

To demonstrate that BDNF-AS recruits WDR5 and promotes the methylation of FBXW7, we predicted the location of CpG islands in the promoter region of FBXW7 by MethPrimer2.0 (Supplementary figure-1M). We then measured the levels of CpG island methylation using a Q-MSP assay. The results suggested that the levels of CpG island methylation were significantly increased after BDNF-AS and WDR5 were both overexpressed compared with other treatments, and the average methylation rate reached up to 74.86%. However, when the expression of both BDNF-AS and WDR5 was knocked down, the CpG island methylation rate was decreased to 19.76% (Fig. 6A). The results showed that the binding of BDNF-AS to WDR5 resulted in significant methylation of the FBXW7 promoter region.

Next, western blotting was performed to screen for histone lysine methylation modification markers, including H3K9me2, H3K9me3, H3K27me2 and H3K27me3, which are associated with transcriptional inhibition^35^. We found that the protein expression levels of H3K27me3 significantly changed when WDR5 was overexpressed or knocked down (Fig. 6B). In addition, combined knockdown or overexpression of BDNF-AS and WDR5 resulted in obvious changes in the protein level of H3K27me3 (Fig. 6C). To investigate whether BDNF-AS could directly suppress the transcription of FBXW7 and facilitate the formation of H3K27me3 in its promoter region by recruiting WDR5, ChIP was performed with BDNF-AS-overexpressing cells. The results indicated that the level of anti-H3K27me3 was significantly increased compared with that of IgG according to qRT-PCR. The qRT-PCR products were identified by agarose gel electrophoresis (Fig. 6D) (primer information: Supplementary table 1). Moreover, after the overexpression or knockdown of WDR5 in BDNF-AS knockdown or overexpression cells, we detected the changes in the FBXW7 and H3K27me3 protein levels. The results showed that the protein expression of FBXW7 was significantly increased after both BDNF-AS and WDR5 were knocked down, while the protein level of H3K27me3 was obviously decreased. When WDR5 was overexpressed in BDNF-AS knockdown cells and WDR5 expression was downregulated in BDNF-AS-overexpressing cells, the changes in FBXW7 and H3K27me3 protein expression were partially reversed (Fig. 6C). The above research data demonstrated that BDNF-AS performed its crucial biological functions by binding to WDR5. Furthermore, the western blotting results revealed that the degree of VDAC3 ubiquitination was the highest in the BDNF-AS knockdown and FBXW7 overexpression groups. The degree of VDAC3 ubiquitination was the lowest in the FBXW7 knockdown and WDR5 overexpression groups. However, after WDR5 knockdown, the level of VDAC3 ubiquitination was partially recovered (Fig. 6E). In conclusion, the above results demonstrated the presence of the BDNF-AS/WDR5/FBXW7 axis, which can regulate ferroptosis in GC PM by regulating VDAC3 protein ubiquitination.

### BDNF-AS facilitated GC formation and PM by regulating ferroptosis *in vitro*

**Figure 7:**
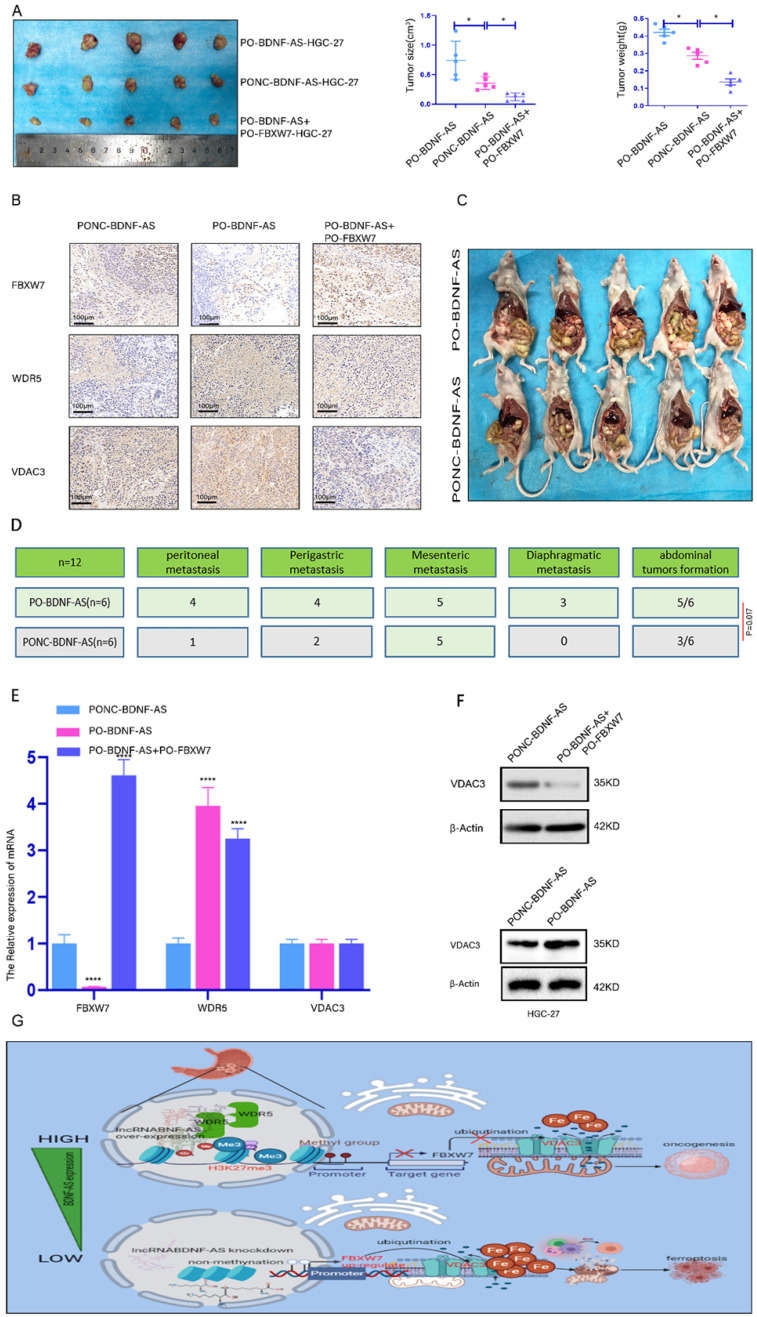
lncRNA BDNF-AS could promote gastric cancer tumorigenesis and peritoneal metastasis *in vivo* . Representative images of migratory or invaded cells were shown. *p < 0.05, **p < 0.01, ***p < 0.001, ****p < 0.0001. Data were shown as mean ± SEM (n = 3). (A): The morphological properties of tumor subcutaneous xenograft, tumor size and tumor weight in PO-BDNF-AS-HGC-27 cells, PONC-BDNF-AS-HGC-27 cells, and PO-BDNF-AS+PO-FBXW7-HGC-27 cells at 30 days, each group had five mice. **(B):** IHC analyzed the expression of FBXW7, WDR5, and VDAC3 proteins of tumors from the PO-BDNF-AS-HGC-27 cells, PONC-BDNF-AS-HGC-27 cells, and PO-BDNF-AS+PO-FBXW7-HGC-27 cells groups. **(C-D):** The image of intraperitoneal tumor formation model from PO-BDNF-AS-HGC-27 and PONC-BDNF-AS-HGC-27 cells at five weeks (C). Simultaneously we counted and analyzed the number of the metastases in peritoneal, perigastric, mesenteric and diaphragmatic (D), each group had six mice. **(E):** The mRNA relative expression levels of FBXW7, WDR5, and VDAC3 of tumors were detected by qRT-PCR assay from the PO-BDNF-AS-HGC-27 cells, PONC-BDNF-AS-HGC-27 cells, and PO-BDNF-AS+PO-FBXW7-HGC-27 cells groups. **(F):** The protein expression level of VDAC3 was analyzed by western blotting from the PO-BDNF-AS-HGC-27 cells, PONC-BDNF-AS-HGC-27 cells, and PO-BDNF-AS+PO-FBXW7-HGC-27 cells groups. **(G):** Proposed the mechanism model in which BDNF-AS/WDR5/FBXW7 axis mediates ferroptosis in GC PM by regulating VDAC3 protein.

We further investigated the role of BDNF-AS and FBXW7 in the oncogenesis and metastasis of GC. We conducted animal experiments with a subcutaneous xenograft model and an intraperitoneal tumor formation model. For the subcutaneous xenograft model, PO-BDNF-AS-HGC-27 cells, PONC-BDNF-AS-HGC-27 cells, or PO-BDNF-AS+PO-FBXW7-HGC-27 cells were injected into the flanks of female nude mice. The neoplasms formed by cells overexpressing BDNF-AS were significantly larger and heavier than those formed by cells in the other groups. Among these results, overexpressing FBXW7 in BDNF-AS-overexpressing cells partially inhibited the tumor formation induced by BDNF-AS overexpression (Fig. 7A). Furthermore, immunohistochemistry was used to detect the protein expression of FBXW7, WDR5, and VDAC3 in the tumors of the three groups. The results indicated that the expression of FBXW7 was significantly reduced in the PO-BDNF-AS group, while the WDR5 and VDAC3 protein expression levels were dramatically increased in the PO-BDNF-AS group. However, we also found that overexpression of FBXW7 could partially reverse the tumorigenic effect of PO-BDNF-AS (Fig. 7B). For the intraperitoneal tumor formation model, PO-BDNF-AS-HGC-27 cells or PONC-BDNF-AS-HGC-27 cells were injected into the lower right abdominal cavity of each mouse. After five weeks of injection, we euthanized the mice and dissected the abdominal cavity to examine tumor formation. As a result, we observed increased abdominal invasion, including PM (4/6 mice), perigastric metastasis (4/6 mice), mesenteric metastasis (5/6 mice), and diaphragmatic metastasis (3/6 mice), after BDNF-AS overexpression. However, in the PONC-BDNF-AS-HGC-27 cell group, we found that abdominal invasion, including PM (1/6 mice), perigastric metastasis (2/6 mice), mesenteric metastasis (3/6 mice), and diaphragmatic metastasis (0/6 mice), occurred in six mice. The results of the chi-square test between the two groups showed that the difference was statistically significant (P=0.017) (Fig. 7C, 7D). These results demonstrated that GC cells were more prone to PM and tumor formation after the overexpression of BDNF-AS. Furthermore, the mRNA expression of FBXW7 was decreased and that of WDR5 was increased in BDNF-AS-overexpressing tumor tissues, but VDAC3 expression was not significantly changed (Fig. 7E). However, the protein expression of VDAC3 was increased in the BDNF-AS-overexpressing tissue compared with the control tissue. In contrast, the protein level of VDAC3 was partly decreased when FBXW7 was overexpressed, indicating that VDAC3 protein expression was regulated at the posttranscriptional level (Fig. 7F). Taken together, these data indicated that BDNF-AS could facilitate GC formation and PM by regulating FBXW7 expression. The schematic of the findings from this current study is presented in Fig. 7G.

## Discussion

BDNF-AS, a natural antisense lncRNA of brain-derived neurotrophic factor (BDNF), is located at chr11:p14.1^36^ and is related to many human cancers, such as colorectal cancer 37 and breast cancer 17. In the present study, we confirmed the function and underlying mechanism of BDNF-AS in GC PM. Microarray analysis was performed to identify differentially expressed lncRNAs in three pairs of PM tissues and primary GC lesion tissues. In our microarray data, we found that BDNF-AS expression was significantly upregulated in PM tissues compared with primary foci (P=0.042). Zhi et al. confirmed that lncRNA BDNF-AS could suppress the proliferation and migration of colorectal cancer by epigenetically repressing GSK-3β expression^37^. Lin et al. illustrated that BDNF-AS plays a major regulatory role in endocrine resistance and malignant progression of breast cancer by regulating the RNH1/TRIM21/mTOR axis through ubiquitination^18^. These studies showed that the expression level of BDNF-AS was different in multiple types of cancers. However, no study has determined the role of BDNF-AS in GC and PM of GC.

LncRNAs play an important role in the processes of genomic transcription, translation and posttranslational modification 38, 39. Moreover, previous studies have suggested that lncRNAs are involved in a variety of biological behaviors of GC, including proliferation, invasion, and metastasis 40-42. Herein, we systematically demonstrated the role of BDNF-AS in PM of GC. Clinically, we showed that BDNF-AS was significantly overexpressed in GC tissues, and its overexpression was associated with the progression, prognosis and PM of patients with GC. *In vitro* studies confirmed that overexpression of BDNF-AS could promote the proliferation, migration and invasion ability of GC cells. Consistent with our study, Lin et al. confirmed that overexpression of BDNF-AS could promote the progression of breast cancer and then lead to poor prognosis^18^. By observing the changes in mitochondria with transmission electron microscopy, we found that GC cell ferroptosis was reduced when BDNF-AS was overexpressed. When BDNF-AS expression was knocked down, ferroptosis was significantly increased. The morphological changes of mitochondria in GC cells were consistent with the findings of previous studies^29^, ^43^, ^44^. Thus, our results indicated that BDNF-AS expression was correlated with the ferroptosis of GC cells. Currently, there is no relevant study on BDNF-AS regulating ferroptosis in the PM of GC, and the underlying mechanism remains unclear. Herein, protein profiling was performed with three pairs of GC tissues and GC PM tissues to explore which proteins were associated with GC PM and ferroptosis; the results suggested that the VDAC3 protein, located in the outer membrane of mitochondria and associated with ferroptosis, was detected. Our further studies confirmed that VDAC3 expression was upregulated in GC tissues and GC PM tissues. Previous studies indicated that VDAC3 could affect cell ferroptosis by regulating the mitochondrial entry and exit of iron ions^19^, ^20^, ^45^. Excessive iron accumulation could result in ROS production through the Fenton reaction and then cause ferroptosis^46^, ^47^. Our results showed that when BDNF-AS was overexpressed, the VDAC3 protein expression level increased. However, the protein level of VDAC3 was decreased when BDNF-AS expression was knocked down. These results indicated that BDNF-AS expression was significantly associated with VDAC3 expression. However, how BDNF-AS regulates VDAC3 expression has not yet been studied.

Studies have proven that approximately 80%-90% of the proteins involved in cell function are degraded through the ubiquitin-proteasome pathway and that ubiquitination is associated with ferroptosis^8^, ^48^. Our further studies showed that VDAC3 expression was significantly correlated with FBXW7 expression. Moreover, Zhu et al. confirmed that autophagy regulated erastin-induced ferroptosis via the FBXW7-VDAC3 axis in acute lymphoblastic leukemia^49^. Ye et al. also showed that the FBW7-NRA41-SCD1 axis could synchronously regulate apoptosis and ferroptosis in pancreatic cancer cells^25^. Our results demonstrated that FBXW7 could regulate VDAC3 protein expression through ubiquitination in GC cells. These studies demonstrated that FBXW7 played a crucial role in regulating VDAC3 protein expression during the process of ferroptosis in PM of GC. Based on an existing study, BDNF-AS could regulate protein degradation through ubiquitination^18^. We hypothesized that BDNF-AS might regulate the expression of VDAC3 by regulating FBXW7 expression to control GC ferroptosis. Emerging research data suggest that lncRNAs can induce gene activation or repression through chromatin modification^34^, ^50^. CpG island methylation in the promoter region of tumor suppressor genes is one of the main chromatin modifications in human tumors^35^. In pancreatic cancer, Jin et al. also suggested that CDK5/FBXW7 could inhibit pancreatic cancer cell migration and invasion by regulating H3K27me3^51^. In our study, we showed that the mRNA and protein expression levels of FBXW7 were negatively correlated with both BDNF-AS and WDR5 expression levels. Moreover, we also demonstrated that the CpG islands in the promoter region of FBXW7 were significantly methylated and that the H3K27me3 protein was obviously enriched when BDNF-AS and WDR5 were overexpressed. Taken together, these results revealed that BDNF-AS regulated the transcription of FBXW7 by recruiting WDR5 to methylate the CpG island of its promoter. When the VDAC3 protein could not be degraded normally, the original dynamic homeostasis process was disrupted, and the abnormal increase in ion and energy metabolism promoted the proliferation, invasion and metastasis of tumor cells, thereby resulting in resistance to ferroptosis^19^, ^21^. *In vivo,* we observed that peritoneal carcinoma was more likely to occur when BDNF-AS was overexpressed. However, when FBXW7 was overexpressed in the BDNF-AS-overexpressing mouse model, ferroptosis was increased, and the carcinogenic effect of BDNF-AS was partially reversed. This evidence indicated that BDNF-AS could negatively regulate FBXW7 expression in PM of GC. Taken together, these data indicated that the levels of BDNF-AS, WDR5, and FBXW7 were closely associated with each other and that these molecules interdependently perform significant biological functions. For the first time, we demonstrated that the BDNF-AS/WDR5/FBXW7 axis could control ferroptosis in PM of GC by regulating the ubiquitination-mediated degradation of the VDAC3 protein. These discoveries provide a theoretical basis for the prognostic evaluation and treatment of PM in GC patients.

In summary, we found that the BDNF-AS/WDR5/FBXW7 axis could regulate the expression of VDAC3, thus regulating ferroptosis in PM of GC. Therefore, BDNF-AS might be a novel and promising therapeutic target and biomarker for patients with advanced GC or GC PM.

## Supplementary Material

Supplementary figure and table.

## Figures and Tables

**Table 1:** the correlation of BDNF-AS expression with the clinicopathological parameters of GC patients (n=66).

Parameters	n(%)	BDNF-AS expression		P value
		Low	High	
Gender				
Male	47(71.2)	16	31	0.312
Female	19(28.8)	9	10	
Age				
≥60 years	43(65.2)	18	25	0.362
<60 years	23(34.8)	7	16	
Cancer grade				
Moderate/well	11(16.7)	6	5	0.212
poor	55(83.3)	19	36	
Cancer size(cm)				
≥5	21(31.8)	6	15	0.287
<5	45(68.2)	19	26	
T-Stage				
T1-2	12(18.2)	7	5	0.106
T3-4	54(81.8)	18	36	
Lymphatic Metastasis				
N0-1	31(47.0)	17	14	0.008
N2-3	35(53.0)	8	27	
Distant Metastasis				
Yes	8(11.67)	0	8	0.018
No	58(88.33)	25	33	
TNM-Stage				
Ⅰ/Ⅱ	22(33.3)	13	9	0.012
Ⅲ/Ⅳ	44(66.7)	12	32	
CEA(ng/ml)				
≥5	13(19.7)	4	9	0.555
<5	53(80.3)	21	32	
CA199(u/ml)				
<37	53(80.3)	22	31	0.220
≥37	13(19.7)	3	10	
Chemotherapy				
Yes	55(83.3)	22	33	0.427
NO	11(16.7)	3	8	
PM				
Yes	20(30.3)	3	17	0.012
No	46(69.7)	22	24	

Remark: Tumor Staging Guidelines: AJCC Cancer Staging Manual (8^th^). Abbreviations: TNM: tumor-node-metastasis, CEA: carcinoembryonic antigen, CA19-9: carbohydrate antigen 19-9, T-Stage: Tumor invasion stage. PM: Peritoneal Metastasis. P < 0.05: statistically significant.

**Table 2:** Univariate and multivariate analyses of clinicopathologic parameters associated with progress free survival and overall survival

Parameters	Overall survival						Progress Free survival					
	Univariate analysis			Multivariate analysis			Univariate analysis			Multivariate analysis		
	HR	95%CI	P	HR	95%CI	P	HR	95%CI	P	HR	95% CI	P
Gender (Male Vs Female)	0.801	0.422-1.521	0.498				0.818	0.431-1.555	0.54			
Age (≥60 years VS <60years)	0.944	0.520-1.713	0.849				0.945	0.520-1.715	0.851			
Grade (poor Vs Moderate/well)	2.119	0.837-5.366	0.113				2.184	0.862-5.530	0.099			
Cancer size (cm) (≥5 VS <5)	2.003	1.100-3.644	0.023	2.086	0.915-4.675	0.081	1.92	1.055-3.494	0.033	1.774	0.792-3.972	0.163
T-Stage (T3-4 VS T1-2)	3.628	1.293-10.181	0.014	1.197	0.239-6.004	0.827	3.671	1.309-10.298	0.013	1.482	0.294-7.471	0.634
LNM (N2-3 VS N0-1)	2.822	1.517-5.252	0.001	2.477	1.099-5.584	0.029	3.509	1.784-6.902	<0.001	2.222	0.999-4.943	0.050
DM (Yes VS No)	6.479	2.767-15.168	<0.001	4.642	1.679-12.837	0.003	2.806	1.507-5.223	0.001	5.721	1.959-16.707	0.001
TNM-Stage (Ⅲ/Ⅳ VS Ⅰ/Ⅱ)	4.009	1.846-8.705	<0.001	0.943	0.274-3.244	0.926	4.029	1.856-8.744	<0.001	0.843	0.244-2.919	0.788
CEA (ng/ml) (≥5 VS <5)	1.414	0.700-2.854	0.334				1.376	0.682-2.776	0.372			
CA199 (u/ml) (≥37 VS <37)	2.364	1.213-4.609	0.012	1.668	0.675-4.120	0.267	2.182	1.119-4.255	0.022	1.170	0.476-2.880	0.732
BDNF-AS expression (high VS low)	5.155	2.444-10.874	<0.001	4.036	1.663-9.796	0.002	5.129	2.434-10.806	<0.001	3.989	1.629-9.767	0.002
Chemotherapy (No VS Yes)	2.611	1.290-5.283	0.008	2.644	1.014-6.891	0.047	2.686	1.325-5.444	0.006	2.986	1.129-7.893	0.027
PM (Yes VS No)	4.451	2.339-8.472	<0.001	3.593	1.625-7.946	0.002	4.753	2.495-9.052	<0.001	4.324	1.959-9.544	0<001

Remark: Tumor Staging Guidelines: AJCC Cancer Staging Manual (8^th^). Abbreviations: TNM: tumor-node-metastasis, CEA: carcinoembryonic antigen, CA19-9: carbohydrate antigen 19-9, T-Stage: Tumor invasion stage. P: P value, PM: Peritoneal Metastasis. P<0.05: statistically significant.

## Abbreviations

GC: Gastric cancer
BDNF-AS: Long noncoding RNA BDNF-AS
VDAC2: voltage-dependent anion channels 2
VDAC3: voltage-dependent anion channels 3
SCF: SKIP-CUL-F-box protein
NC: Negative control
qRT-PCR: Quantitative reverse transcription polymerase chain reaction
WB: western blotting
PM: Peritoneal Metastasis
PMT: Peritoneal Metastasis tissue
LNM: lymph node metastasis
TNM: tumor-node-metastasis
DM: Distant Metastasis
CA19-9: carbohydrate antigen 19-9
CEA: carcinoembryonic antigen
IHC: Immunohistochemistry
MDA: Malondialdehyde
GSH: Glutathione
GSSG: glutathione disulfide
Q-MSP: quantitative methylation specific PCR
IP: immunoprecipitation
CO-IP: Co-Immunoprecipitation
RIP: RNA-Binding Protein Immunoprecipitation
ChIP: Chromatin immunoprecipitation
ChIRP: Chromatin isolation by RNA purification Chromatin isolation by RNA purification
Lv-Oe-BDNF-AS: lentivirus mediated over-expression of BDNF-AS
Lv-ON-BDNF-AS: lentivirus mediated negative control of BDNF-AS
PO-BDNF-AS: pCDNA-over-expression of BDNF-AS
PN-BDNF-AS: pCDNA-nagative control of BDNF-AS
Lv-SH-BDNF-AS: lentivirus mediated knockdown of BDNF-AS
Lv-SHNC-BDNF-AS: lentivirus mediated negative control of BDNF-AS
Lv-Oe-FBXW7: lentivirus mediated over-expression of FBXW7
Lv-ON-FBXW7: lentivirus mediated negative control of FBXW7
PO-FBXW7: pCDNA-overexpression of FBXW7
SH-FBXW7: pCDNA-knockdown of FBXW7
SHNC-FBXW7: pCDNA-knockdown negative control of FBXW7
