# Supplementary material for: The lncRNA BDNF-AS/WDR5/FBXW7 axis mediates ferroptosis in gastric cancer peritoneal metastasis by regulating VDAC3 ubiquitination
Source: Int J Biol Sci. 2022 Jan 24;18(4):1415–33. doi: 10.7150/ijbs.69454 (PMC8898362; doi:10.7150/ijbs.69454)

Supplementary table-1: Related gene primers mentioned in this article

Gene	sequence
lncRNABDNF-AS	F:5'-CCGTGAGAAGATCTCATTGGGC-3′.<br>R:5'-TGGGTCACAAGTCACGTAGC-3′.
lncRNABDNF-AS FISH probe sequence (5'-3')	cttaag+tgagcctaag+atacatttaga+tgaatttgt gc+tgttgtttcaagaggc+ttgacatca+ttg.
lncRNABDNF-AS + WDR5-CHIP-primers	
Site1	F1:5'-ACAGTCCCCATGTATCGGTC-3'.<br>R1:5'-TGCAATGGAAGCAAATAGACCTT-3'(71bp).
Site2	F2:5'-TGGGAAGACTTGCCGAGTT-3'.<br>R2:5'-ACCAAACAGATTTGCTCCCATC-3'(94bp).
Site3	F3:5'-AAGGGTTCGAAGCGATTGGG-3'.<br>R3:5'-CTTGCGGCTTTTTAGGTCGG-3'(140bp).
lncRNABDNF-AS + H3K4me27-CHIP-primers	
Site1	F1:5'-CCGAATACAAGCGAGGTT-3'.<br>R1:5'-GCACAGACTACACTCATTG-3'(129bp).
Site2 (paper use)	F2:5'-CAGGCAATGAGTGTAGTCT-3'.<br>R2:5'-AAGGCACAGCACAACTAA-3'(156bp).
Site3	F3:5'-TTAGTTGTGCTGTGCCTTG-3'.<br>R3:5'-TCTCTGTGCCTGGCTCTA-3'(100).
FBXW7	F:5'-GTGGGACATACAGGTGGA-3′.<br>R:5'-CAACGCACAGTGGAAGTA-3′.
FBXW7 knockdown plasmid sequences	
FBXW7-RNAi(71227-1)	TGGAATGGAACTCAAAGACAA.
FBXW7-RNAi(71228-1)	CCATGCAAAGTCTCAGAATAT.
FBXW7-RNAi(71229-1)	ACCTACAGACTAAGAAGGAAA.
WDR5:	F: 5'-AATATCCGATGTAGCCTGGTC-3'.<br>R: 5'-TTGGACTGGGGATTGAAGTTG-3'.
METTL3	F: 5'-CCAGCACAGCTTCAGCAGTTCC-3'.<br>R: 5'-GCGTGGAGATGGCAAGACAGATG-3'.
SETD3	F: 5'-GACAGACTCTACGCCATG-3'.<br>R: 5'-GTCTCCCAGCAAGTGTTC-3'.
KMT2C	F: 5'-AAGCAAACGGACTCAGAGGA-3'.<br>R: 5'-ACAAGCCATAGGAGGTGGTG-3'.
KMT2D	F: 5'-AGCCTCCGCACCAACAATC-3'.<br>R: 5'-AGCTCCTCGCCCTTCAGATA-3'.
WDR5 overexpression plasmid sequences	
pCDNA-WDR5-1	CACACTGGACTAGTGGATCCCGCCACCATGGCG+ ACGGAGGAGAAGAAG.

pDNA-WDR5-2	AGTCACTTAAGCTTGGTACGCAGTCACTCTTCC+ ACAGTTTAATTG.
VDAC3	F:5'-CTTGGGAGAATAAGTTGGCTGAA-3'. R:5'-AAGTCCGCAGCCTTGTAACC-3'.
WDR5-interference sequences	
Si-WDR5-1	GCUCAGAGGAUAACCUUGUTT+ ACAAGGUUAUCCUCUGAGCTT
Si-WDR5-2	GGAUCCUUGAUAGUUUUCAATT+ UUGAAACUAUCAAGGAUCCTT
β-actin	F:5'-GACCACCTTCAACTCCATCAT-3'. R:5'-CCTGCTTGCTAATCCACATCT-3′.
U6	F:5' -CTCGCTTCGGCAGCACA-3' R:5' -CTCAACTGGTGTCGTGGA-3'
GAPDH:	F:5' -GGAAGCTTGTCATCAATGGAAATC-3'. R:5' -TGATGACCCTTTTGGCTCCC-3'.
lncRNABDNF-AS CHIRP primers	
lnc40024687-NC	TACGCACCACTAGTCGTACT-/3bio/
lnc40024688	ATACAAGGGCAGGAAGGAGA-/3bio/
lnc40024689	TGAGCCTAAGATACATTGCT-/3bio/
lnc40024690	CGTGATAGAAGAGCTGTTGG-/3bio/
lnc40024691	GGCAGGTTCAAGAGGCTTGA-/3bio/
lnc40024692	AAACAGGATGGCTCAATGAA-/3bio/
lnc40024693	CCTTGGGCTCATCTCTACAG-/3bio/
lnc40024694	GATTTCCAGATAAACAAGGC-/3bio/
lnc40024695	AGGGAGCCAACAAACAACTG-/3bio/
lnc40024696	TTGGCTGACACTTTCGAACA-/3bio/
lnc40024697	ATAGTAGGTCAACATTGGTG-/3bio/
lnc40024698	AGCCTTTCATAGCCCATTCA-/3bio/
lnc40024699	AGGGAGCCAACAAACAACTG-/3bio/
FBXW7 promoter-Q-MSP-primers	
ME039-S5-MF	F:5'-CGAGTTTGAGTCGGAGTCGGC-3'.
ME039-S5-MR	R:5'-CGAAACGAAACAAAAACTCTAACG-3'.
ME039-S5-UF	F:5'-GTTTGAGTTTGAGTTGGAGTTGGT-3'.
ME039-S5-UR	R:5'-CACAAAACAAAACAAAAACTCTAACA-3'.

Supplementary figure 1

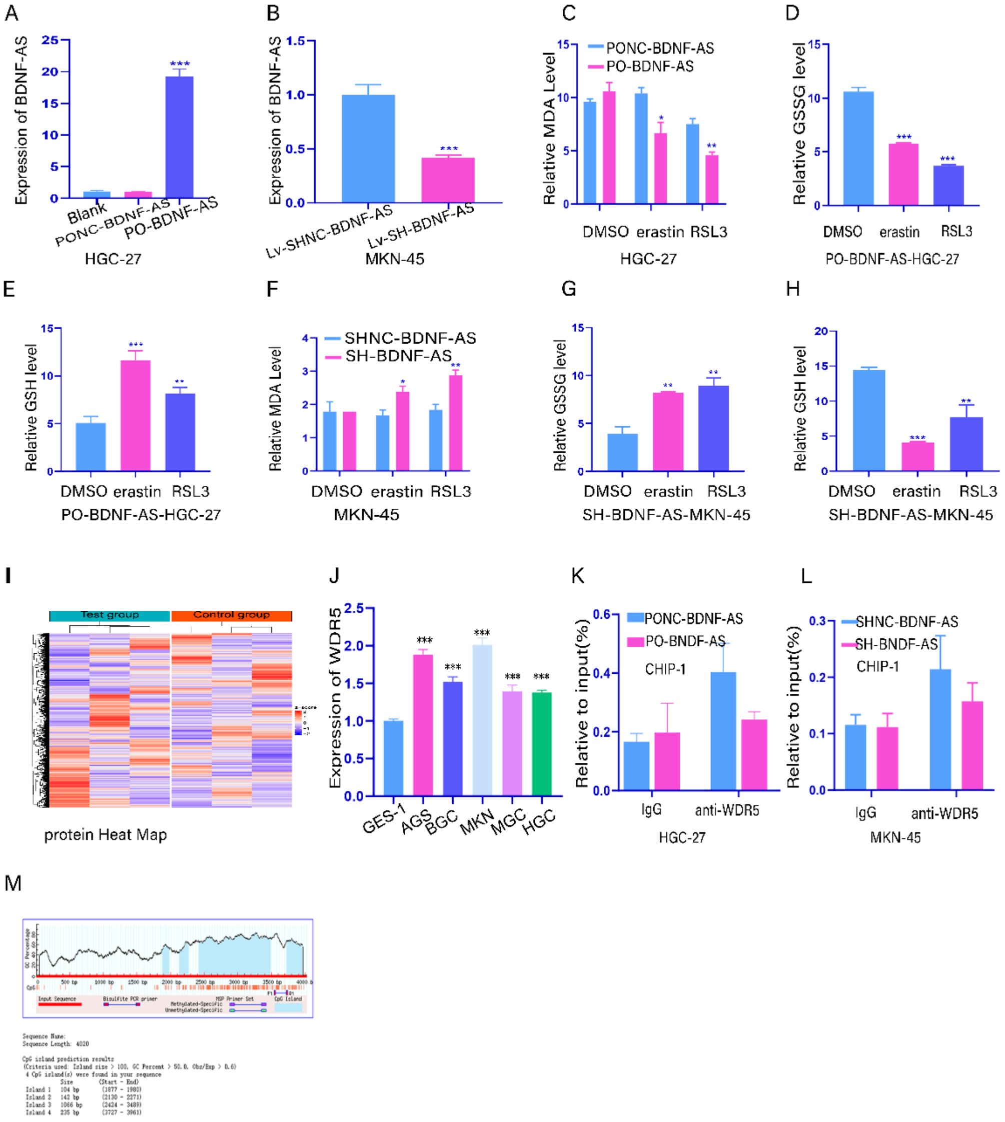

Supplement: Supplementary file 1 — Supplementary figure and table. [file ijbsv18p1415s1.pdf]
